# Pneumonia among Children Admitted to the Department of Medicine in a Tertiary Care Centre: A Descriptive Cross-sectional Study

**DOI:** 10.31729/jnma.7859

**Published:** 2022-09-30

**Authors:** Raj Kumar B.K., Swesha Shrestha, Siddhant Adhikari, Shristi Maharjan

**Affiliations:** 1 Kanti Children Hospital, Maharajgunj, Kathmandu, Nepal; 2 Grande City Hospital, Kantipath, Kathmandu, Nepal; 3 Motherland Hospital, Pepsicola, Kathmandu, Nepal; 4 Karuna Hospital, Budhanilkantha, Kathmandu, Nepal

**Keywords:** *paediatrics*, *pneumonia*, *prevalence*, *tertiary care centers*

## Abstract

**Introduction:**

Pneumonia is one of the most common infectious causes of death in children around the world, accounting for 14% of all deaths of children under five years of age. The study aimed to find out the prevalence of pneumonia among children admitted to the Department of Medicine in a tertiary care hospital.

**Methods:**

A descriptive cross-sectional study was conducted in the Department of Medicine of paediatrics tertiary care centre from 1 July 2021 to 30 June 2022 among children aged 2-59 months. Ethical approval was obtained from the Institutional Review Committee (Reference number: 94). Convenience sampling method was used. Data were collected from hospital records during the study period. Point estimate and 95% Confidence Interval were calculated.

**Results:**

Among 385 children, pneumonia was seen in 76 (19.74%) (15.76-23.72, 95% Confidence Interval) children. A total of 30 (39.47%) patients were in the age group of 2-11 months, 52 (68.42%) were males, 38 (50%) required O_2_ supplementation, 26 (34.21%) required transfer to the Intensive Care Unit, and 53 (69.74%) patients stayed for <7 days in the hospital.

**Conclusions:**

The prevalence of pneumonia in children admitted to the Department of Medicine was found to be higher than similar studies conducted in similar settings, with higher prevalence in young infants and the male sex than other age groups and genders, respectively.

## INTRODUCTION

Pneumonia is an infection of the lung or pulmonary parenchyma. There are more than 1,400 cases of pneumonia per 100,000 children, or 1 case per 71 children globally.^1^ It is one of the most common infectious causes of death in children around the world, accounting for 14% of all deaths of children under five years of age.^2^

In a resource-limited country like Nepal, among total acute respiratory infection (ARI) cases, 12.48% were identified as having pneumonia, with a case fatality rate of 0.13 per 1000.^3^ Several risk factors such as malnutrition, low birth weight, non-exclusive breastfeeding, overcrowding at home, and the use of polluting cooking fuel have been identified as the major contributors to childhood pneumonia in low and middle-income countries (LMICs).^4^ Establishing the prevalence of pneumonia and determining its outcome is important for proper planning and interventions.

This study aimed to determine the prevalence of pneumonia among children admitted to Department of Medicine in a tertiary care centre.

## METHODS

A descriptive cross-sectional study was conducted among children admitted to a Kanti Children Hospital in the Department of Medicine between 1 July 2021 to 30 June 2022. Ethical approval (Reference number: 94) was obtained from the Institutional Review Committee of the same institute. Data were collected from hospital-based records during this study period. All the children aged 2-59 months admitted to the Department of Medicine of a pediatric tertiary care facility during the study duration were included. Incomplete data, patients discharged on leaving against medical advice (LAMA), and with clear alternative diagnoses were excluded. A convenience sampling method was used. The sample size was calculated using the following formula:


$$\begin{array}{ll} \text{n} & ={\text{Z}}^{2}\times \frac{\text{p}\times \text{q}}{{\text{e}}^{\text{2}}}\\ & =1.96^{2}\times \frac{0.5\times 0.5}{0.05^{2}}\\ & =385 \end{array}$$

Where,

n= minimum required sample size
Z= 1.96 at 95% Confidence Interval (CI)
p= prevalence taken as 50% for maximum sample size calculation
q= 1-p
e= margin of error, 5%

The calculated sample size was 385. Children admitted to the inpatient medical ward with the primary diagnosis of "pneumonia" by the clinicians according to the World Health Organization (WHO) case definition during the study period were included in the study.^5^ The length of the hospital stay was calculated by subtracting the date of admission from the date of discharge. Oxygen supplementation requirement during any time in the course of the treatment was considered. Data regarding the diagnosis, age, gender, socio-demographic characters, and the outcome were collected.

Data were entered in Microsoft Excel 2016. Error and inconsistency were verified after checking the source document and statistical analysis was done using IBM SPSS Statistics 21.0. Point estimate and 95% CI were calculated.

## RESULTS

**Figure 1:**
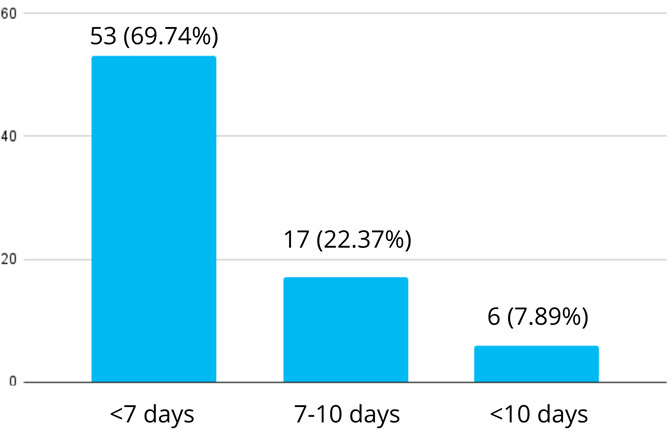
Length of hospital stay among the children aged 2-59 months (n= 76)

Among 385 children, pneumonia was seen in 76 (19.74%) (15.76-23.72, 95% CI). A total of 53 (69.74%) patients stayed for less than 7 days in the hospital, 17 (22.37%) patients stayed for 7-10 days, and six (7.89%) patients stayed for more than 10 days (Figure 1).

O_2_ supplementation was required in 38 (50%) patients and 26 (34.21%) required ICU transfer (Table 1).

**Table 1:** Treatment course in hospital admitted children with pneumonia (n= 76).

Characteristics	n (%)
O_2_ supplementation	38 (50)
ICU transfer	26 (34.21)

The majority of children weighing 2600-3900 grams at birth 43 (56.58%) were affected by pneumonia. Thiry one (40.79%) children had a birth weight ranging from 1500-2500 grams while two (2.63%) children weighed <1500 grams at birth. Thirty six (65.79%) children whose mother's age ranged from 21-30 years had a higher prevalence of pneumonia. Children in a family with 4-6 members were affected more 38 (50%). Thirty six (47.37%) children were the first children and were affected more than any other order child (Table 2).

**Table 2:** Characteristics of the child and their family affected with pneumonia (n= 76).

Characteristics	n (%)
**Mothers’s age (years)**	
<21	11 (14.47)
21-30	50 (65.79)
30-40	15 (19.74)
>40	-
**Family size**
<4	36 (47.37)
4-6	38 (50)
>6	2 (2.63)
**Child order**
1	36 (47.37)
2	25 (32.89)
3	11 (14.47)
4	3 (3.95)
5	1 (1.32)
>6	-

Out of 76 children with pneumonia, 52 (68.42%) were males and 24 (31.58%) were females, with a male to female ratio of 2.16:1. The mean age of children was 19.17 months. Thirty (39.47%) pneumonia cases were found among those aged 2-11 months, followed by 17 (22.37%) cases in 12-23 months age, and the lowest number of five (6.58%) cases was found in 48-59 months (Table 3).

**Table 3:** Distribution of pneumonia according to age and sex among 2-59 months age children (n= 76).

Characteristics	n (%)
**Age group (months)**	
2-11	30 (39.47)
12-23	17 (22.37)
24-35	13 (17.11)
36-47	11 (14.47)
48-59	5 (6.58)
**Sex**	
Male	52 (68.42)
Female	24 (31.58)

## DISCUSSION

A descriptive study of 385 cases of children from 2-59 months hospitalised with pneumonia in the department of medicine was conducted. The prevalence of pneumonia among children aged 2-59 months was found to be 19.74% among the patients admitted to the department of medicine, which is higher compared to the previous study conducted in our country (4.5-7.1%).^6^ This discrepancy could be due to the institutional basis and seasonal variation of the disease. Also, surgical cases and children under 2 months were excluded, and only inpatient admissions to the medicine department were included in our study. However, a study conducted in Ethiopia has shown the prevalence to be 33.5%.^7^ This might be due to the socioeconomic differences from our country.

In our study, the majority of children with pneumonia were infants in the age group of 2-11 months, which is similar to studies conducted in Nepal and Taiwan.^6,8,9^ This may be because they are more susceptible to general infections than other age groups. Our study found that a sizable portion of the burden of pneumonia in children among 2-59 months consisted of males, with a male to female ratio of 2.16:1. Similar findings have been reported in studies from Nepal and Bangladesh.^6,10^ In contrast, one study from our nation reported a higher prevalence in females.^8^

In the present study, those children in a family with 4-6 members were affected more than those in a small family with less than 4 members. This is in contrast to the findings from a study which reported pneumonia to be more common in children in a family with less than 4 members.^11^ The average size of a family in our country is more than four, which could have affected the results of our study.

In our study, 50% of the patients with pneumonia required oxygen supplementation during the course of treatment. A study from Ethiopia showed a higher percentage (74.29%) of patients required oxygen supplementation for the treatment.^12^ A higher percentage could have been reported as patients in respiratory distress are more likely to be admitted and need oxygen therapy in comparison to patients managed on an outpatient basis. 69.74% of the patients had a hospital stay duration of less than 7 days, which is similar to the study done in Bangladesh and Ethiopia.^13,14^

This study has few limitations. It was conducted in an exclusive single hospital setting and the findings may not be generalisable. The severity of pneumonia was not classified which may affect the oxygen supplementation requirements, duration of hospital stay and ICU admissions. Therefore, the study's limitations should be taken into consideration before any application of the findings.

## CONCLUSIONS

The prevalence of pneumonia in children admitted to the medicine department was found to be higher than in similar settings. Pneumonia could impose an immense burden on a vulnerable age group of children aged 2-59 months, with a higher prevalence among 2-11 month old children and those of male sex. The oxygen supplementation requirement among admitted children was high. However, the duration of hospital stay was found to be shorter. Further studies in children with pneumonia including different variables to assess the risk factors and outcome of the disease could be recommended.
